# Forests buffer against variations in precipitation

**DOI:** 10.1111/gcb.15763

**Published:** 2021-07-28

**Authors:** John C. O'Connor, Stefan C. Dekker, Arie Staal, Obbe A. Tuinenburg, Karin T. Rebel, Maria J. Santos

**Affiliations:** ^1^ Copernicus Institute of Sustainable Development Department Environmental Sciences Utrecht University Utrecht The Netherlands; ^2^ Department of Geography University of Zürich Zurich Switzerland

**Keywords:** atmospheric transport, evapotranspiration, forests, moisture recycling, precipitation variability

## Abstract

Atmospheric moisture recycling effectively increases the amount of usable water over land as the water can undergo multiple precipitation–evapotranspiration cycles. Differences in land cover and climate regulate the evapotranspiration flux. Forests can have deep roots that access groundwater facilitating transpiration throughout the dry season independent of precipitation. This stable transpiration buffers the forest against precipitation variability. However, it is not known whether the buffering effect, already modeled for tropical forests, is common to all forests globally. Here we apply a state‐of‐the‐art Lagrangian moisture tracking model (UTrack) to study whether forest land cover in the upwind precipitationshed can lead to a reduction in monthly precipitation variability downwind. We found a significant buffering effect of forests in the precipitation variability of 10 out of 14 biomes globally. On average, if 50% of precipitation originates from forest, then we find a reduction in the coefficient of variation of monthly precipitation of 60%. We also observed that a high fraction of precipitation from non‐forest land sources tends to have the opposite effect, that is, no buffering effect. The average variation of monthly precipitation was 69% higher in areas where 50% of precipitation originates from non‐forest land sources in the precipitationshed. Our results emphasize the importance of land cover composition in the precipitationshed to buffer precipitation variability downwind, in particular forest cover. Understanding the influence of land cover in a precipitationshed on atmospheric moisture transport is key for evaluating an area's water‐climate regulatory ecosystem services and may become increasingly important due to continued changes in land cover and climate change.

## Graphical abstract

Precipitation originates from evapotranspiration in the upwind region known as the precipitationshed. The precipitationshed is unique for each area and can be composed of a combination of ocean, forest and non‐forest land covers. We found that areas with a higher fraction of precipitation originating from forests had lower variability in monthly precipitation while precipitationsheds largely composed of non‐forest land covers had higher variability in precipitation. Our study further demonstrates the important climate regulatory services provided by forests globally.

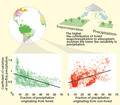

## 1 INTRODUCTION

Atmospheric moisture recycling is a highly important process in the hydrological cycle, as it effectively increases the available water within a catchment and at regional scales (van der Ent et al., 2010). It is the process by which evaporated water is returned to the atmosphere where it can precipitate in situ or be carried downwind and precipitate out (Aragão, 2012). This process of making rain and the redistribution of this moisture are crucial for rain‐dependent processes such as natural ecosystem functioning, crop production and securing water for human well‐being (António Sumila et al., 2017; Keys et al., 2016). Moisture recycling is affected by land‐cover‐driven differences in evapotranspiration or evaporation fluxes, which directly alter the amount of precipitable water downwind (Jasechko et al., 2013; Spracklen et al., 2012). Thus, the land cover type at an evapotranspiration site may influence the magnitude and variability of downwind precipitation. Globally, the average residence time of water in the atmosphere following evapotranspiration is 9 days (van der Ent & Tuinenburg, 2017). We refer to a reduction in precipitation variability by the upwind land cover as “buffering.” Knowing this buffering effect is fundamental to understand the water‐climate regulatory ecosystem service of moisture recycling and assess water security at local, regional and global scales (Keys et al., 2019).

Atmospheric transport of moisture can carry water 100–1000 km before it rains out (Staal et al., 2018; van der Ent & Savenije, 2011). Due to this, a single molecule of water can undergo several precipitation–evapotranspiration cycles before it enters the ocean again (Zemp et al., 2014). The “upwind” source area of evaporation or evapotranspiration that contributes to a specific location's precipitation is known as the precipitationshed (Keys et al., 2012). We use the term evapotranspiration to mean the combined flux of evaporation and transpiration from the land and the term evaporation to mean the flux from the ocean. Land cover within the precipitationshed affects the contribution of water to atmospheric moisture due to differences in the evapotranspiration flux. Land covered by bare soil only contributes to atmospheric moisture through evaporation, which is dependent on solar radiation, wind speed, soil moisture availability of the topsoil and humidity (Black et al., 1969). However, for vegetated land cover types, transpiration (Dekker et al., 2000) and leaf surface area increase interception evaporation (Vrugt et al., 2003). Short vegetation such as grasses and crops have a simple canopy structure and relatively shallow rooting depths, making transpiration strongly dependent on recent precipitation (O'Connor et al., 2019) while trees and forests have taller, more complex canopies that facilitate higher evapotranspiration. Trees also have deeper rooting depth than grasses, which decouples their transpiration from recent precipitation as they can access deeper groundwater stores (Nepstad et al., 1994). At these local scales, access to deeper groundwater reduces the variability of the evapotranspiration flux and protects the forest from drought by maintaining water supply for photosynthesis (Maeda et al., 2017; Nepstad et al., 1994). Studies have shown that, for example, Amazonian forests can maintain evapotranspiration during the dry season (3–4 months) due to their deep roots and access to deep groundwater (Maeda et al., 2017). It has been estimated that evapotranspiration of forested areas in the Amazon ranges from 105.4 to 122.2 mm month^−1^ while grasslands have evapotranspiration ranges from 44 to 108.5 mm month^−1^ (Paca et al., 2019), varying seasonally. Forest transpiration can contribute up to 70% of regional precipitation at the end of the dry season in parts of the Amazon (Staal et al., 2018). Given the ability of trees to tap into deeper water sources and of forest evapotranspiration being a major contributor to atmospheric moisture, not only the Amazon forest but also forests worldwide may contribute to reducing precipitation variability, that is, that forests contribute to a more generalized process of buffering precipitation variability.

As evapotranspiration across the precipitationshed contributes to the atmospheric moisture content and therefore influences the magnitude and variability of precipitation, here we aim to answer how does the land cover within a given precipitationshed affect the variability of precipitation, that is, exerts a buffering effect? Based on previous research in the Amazon, we expect that areas with a high fraction of precipitation originating from forests within its precipitationshed would have lower variability in precipitation than areas with a low fraction of precipitation originating from forests. To test this hypothesis, we conduct a global analysis using a state‐ofthe‐art Lagrangian moisture transport model UTrack (Tuinenburg & Staal, 2020) to calculate the origin of precipitation at a 1° resolution. We then determine the fractions of precipitation originating from forests, non‐forest and oceans, and relate these values to the monthly variability in precipitation. Through these analyses, we determine the contribution of forests to atmospheric moisture and the resulting buffering of precipitation downwind. Such an understanding of these contributions of forest to atmospheriic moisture highlights the interdependency of downwind land cover types on water originating from forests elsewhere, a fundamental ecosystem service at local, regional and global scales.

## 2 METHODS

### 2.1 Model

For our analysis, we utilize the moisture transport model UTrack developed by Tuinenburg and Staal (2020). The model uses a Lagrangian approach to reconstruct atmospheric moisture flows and is forced with the latest and most detailed reanalysis data from ECMWF, ERA5 (Copernicus Climate Change Service, 2017). ERA5 provides hourly global atmospheric data at 0.25^o^ × 0.25^o^ resolution. The model tracks moisture parcels through the atmosphere from evapotranspiration to precipitation at 0.1 h time steps. To achieve this, the hourly ERA5 data are interpolated to 0.1 h increments. Furthermore, data from ERA5's 25 atmospheric layers are re‐distributed within the vertical range of each atmospheric layer to realize as accurate‐as‐possible atmospheric trajectories.

At each time step, evapotranspiration “parcels” equivalent to 0.01mm of water are released and distributed randomly within the atmospheric column. While the magnitude of evapotranspiration is determined by the ERA5 data, the exact location within each grid cell is randomized. The released parcels are tracked through the atmosphere across three dimensions using interpolated ERA5 wind speed and direction. Vertical mixing is simulated using a randomization probabilistic process which redistributes parcels approximately once every 24 h. Finally, precipitation data from ERA5 are used to determine the amount of precipitation at a given location which is removed proportionately from each of the available parcels. Each moisture parcel is tracked until depletion (<1% of tracked moisture remaining) or it has remained within the atmosphere for 30 days. Although this process is more intensive than that in older moisture tracking models, it provides a more complete accounting of moisture flows. As the simulations are forced with ERA5 reanalysis data at all stages, they are supported by on the best available climate data. Furthermore, extensive sensitivity analyses were done for optimal use of the ERA5 reanalysis data. A full explanation of the model including all equations and sensitivity analyses can be found in Tuinenburg and Staal (2020).

During this study, we included an extra label to each “parcel” of water to identify which land cover source the water originates from. We used the land cover data from Song et al. (2018) as it provides the fraction of tall vegetation, short vegetation and bare soil cover updated annually from 1982 to 2016 at a high spatial resolution (0.01°). We used these labeled parcels to calculate the total monthly precipitation originating from three land cover classes: forest ‐ equivalent to tall vegetation, non‐forests ‐ equivalent to the combination of short vegetation and bare soil, and ocean ‐ equivalent to all non‐land areas between 2000 and 2016 rescaled to 1° resolution.

### 2.2 Analysis

The first step was to assess whether there are geographic differences related to the origin of precipitation. We visualized the mean monthly fraction of precipitation originating from the three land cover types calculated from 2000 to 2016 for the global extent. We then computed the fraction of forest cover, the mean monthly precipitation and its coefficient of variation (CV) per biome.

Due to geographic differences in climate, vegetation and precipitation, we decided to conduct our analysis using biomes. We used the ecoregions dataset from Dinerstein et al. (2017) downloaded from https://ecoregions2017.appspot.com. This dataset provides vectorized data of 14 biomes segmented by dominant climate and vegetation, which we rasterized at 1° resolution (Figure S1).

We examined how monthly precipitation is related to forests in the precipitationshed. We conducted a set of regression analyses per biome, where we regressed the CV in precipitation derived from monthly ERA5 data between 2002 and 2017 as a function of the fraction of precipitation originating from forest, non‐forest or ocean in the precipitationshed. If the regression had a negative slope, we interpreted it as the precipitation variability decreasing with an increase in precipitation contribution from that source, that is, a buffering effect. To calculate the precipitation variability of each grid cell, we used the CV in monthly precipitation. We chose the CV because it is a standardized metric, making it possible to compare precipitation variability among locations while accounting for differences in precipitation magnitude. As we use all available cells in a given biome, the relationships from our analysis are not bounded by the assumptions regarding sampling of typical statistical analyses. We present model statistics (F‐test for regression line fit, t‐test for significance of the regression coefficient, confidence intervals for reliability in the estimates of the coefficients) and fit parameters (coefficient of determination—*R* ^2^). All analyses were performed using MATLAB R2018b.

## 3 RESULTS

### 3.1 Fraction of precipitation origin

**FIGURE 1:**
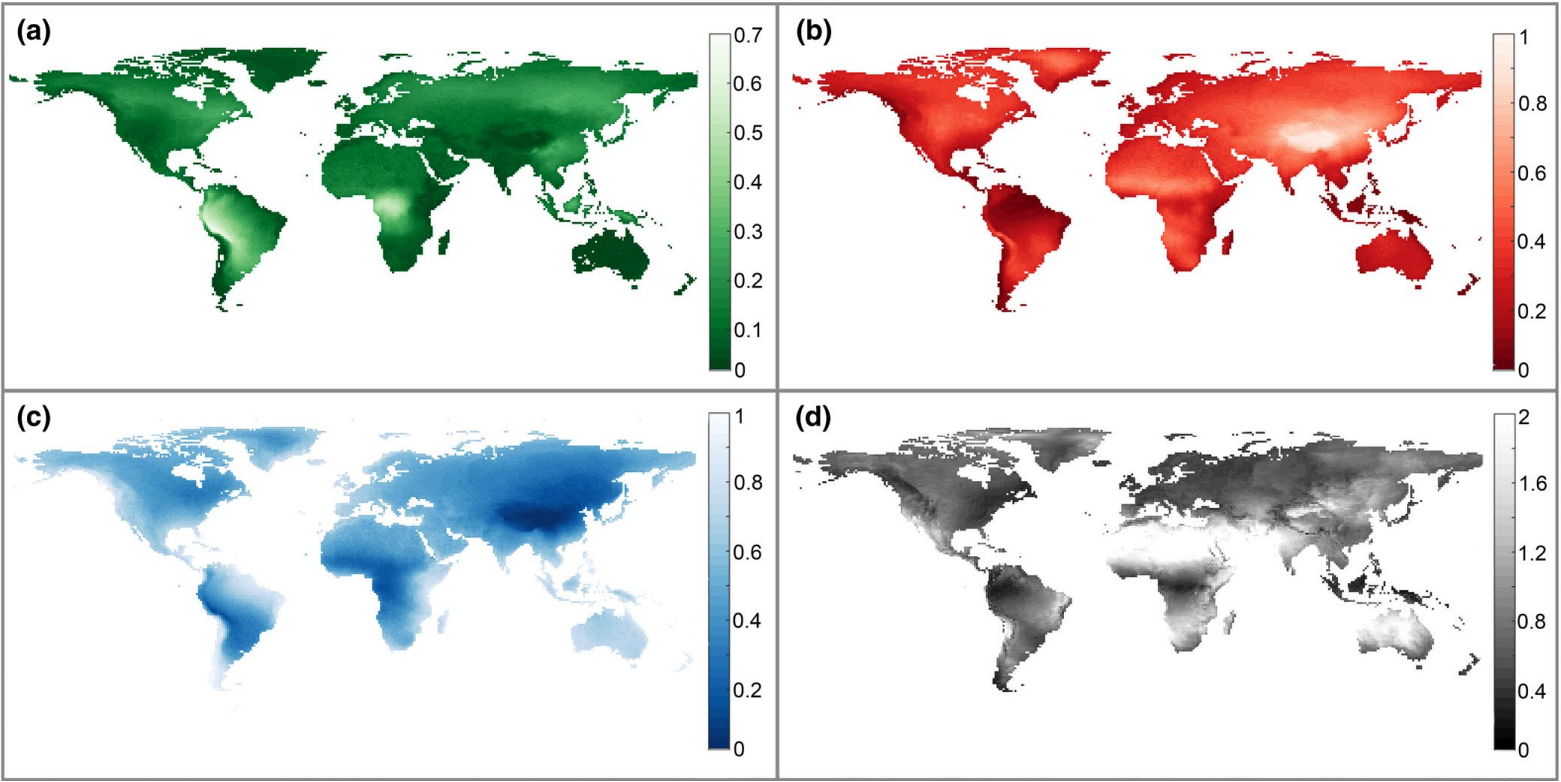
Mean monthly fraction of precipitation by origin: (a) Forests; (b) Non‐forest; (c) Ocean; (d) CV of monthly precipitation. The sum of the first three panels (a–c) is equal to 1 for each 1° grid cell, and this display was chosen to easily identify areas that are more dependent on a particular precipitation source. Note that the range of (a) is 0–0.7. For example, we can see that areas of western South America and the Congo basin rely heavily on precipitation from forests while ocean is the dominant precipitation source for many coastal areas and Australia

We found strong geographic differences in precipitation origin due to forest, non‐forest and oceans (Figure 1). Forest precipitation origin is dominant only in the Amazon (South America) and the Congo (Africa) basins, where these fractions are greater than 0.5 (Figure 1a). Parts of Southeast Asia, eastern Russia and North America have a lower fraction of precipitation that originates from forest, around 0.3 (Figure 1a). We also observe a general pattern of high fractions of ocean origin near coastal areas (Figure 1c) while larger inland landmasses show higher fractions of both forest and non‐forest origins (Figure 1a,b). The highest CV of monthly precipitation was concentrated in dry biomes where there is an extremely low mean monthly precipitation (Figure 1d).

### 3.2 Buffering

#### 3.2.1 Precipitation from forest origin

**FIGURE 2:**
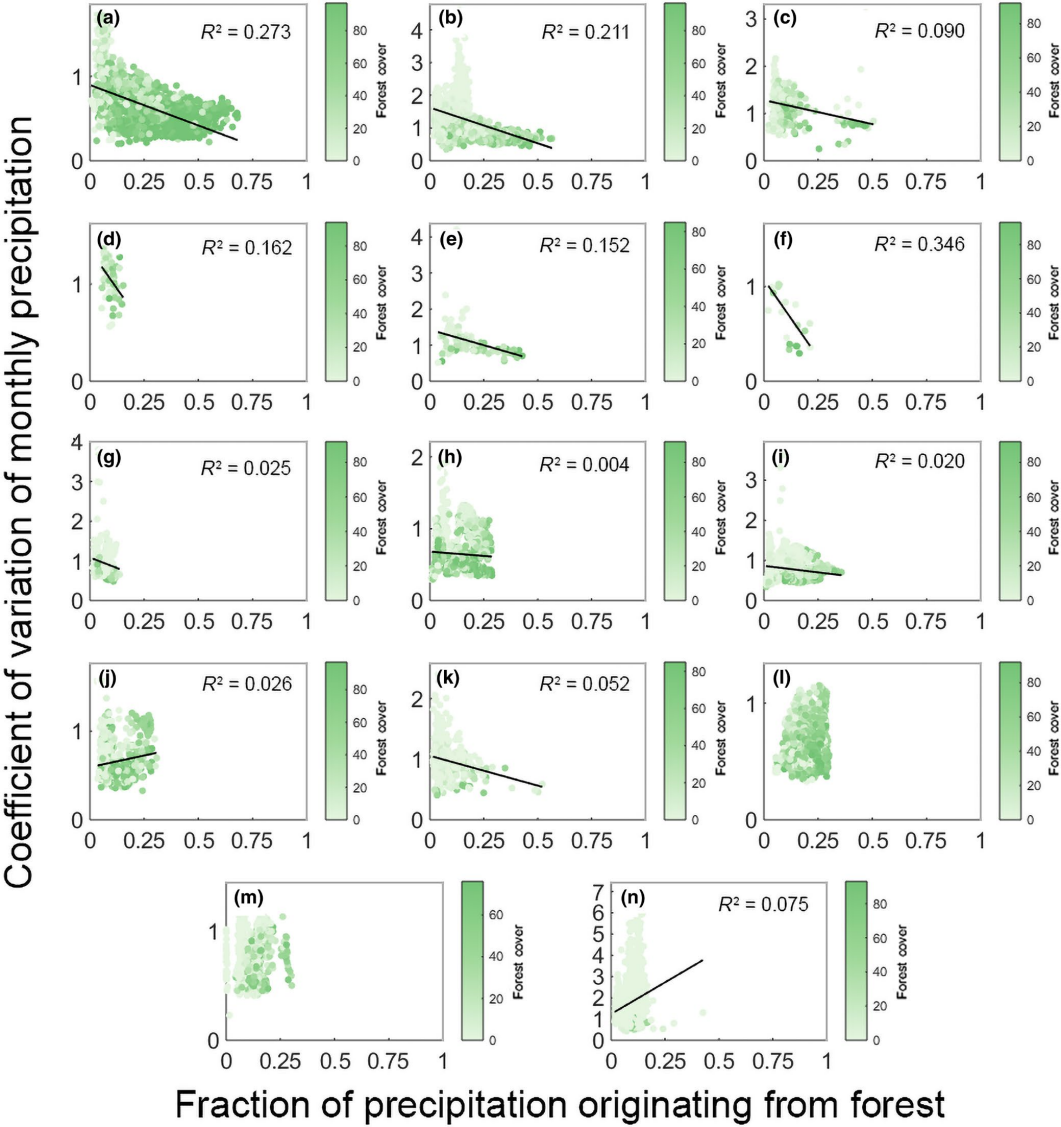
Linear regression of fraction of precipitation originating from forest and coefficient of monthly precipitation variation for 14 biomes globally. Dots in the scatter plot are color coded indicating the fraction of forest cover at sink cell. For plots where significant effects (*p* < 0.05) were detected we include a regression line and the *R* ^2^ value; we do not include a regression line when effects were non‐significant. (a) Tropical moist broadleaf forests; (b) Tropical savannas; (c) Tropical dry broadleaf forests; (d) Tropical coniferous forests; (e) Flooded Grasslands; (f) Mangroves; (g) Mediterranean forests; (h) Temperate broadleaf forests; (i) Temperate grasslands; (j) Temperate coniferous forests; (k) Montane grasslands; (l) Boreal forests; (m) Tundra; (n) Deserts

We found a significant negative effect of the fraction of precipitation originating from forest on the CV of monthly precipitation for 10 of the 14 biomes analyzed (Figure 2; Table S2). This means that, in these biomes, forests have a buffering effect, as areas that have a higher fraction of precipitation originating from forest within their precipitationshed have a lower variability of monthly precipitation. On the other hand, the areas with lower fractions of precipitation originating from forest have higher variability in precipitation. In other words, for 10 out of 14 biomes globally, forests are found to have a buffering effect against precipitation variability.

The strongest buffering effect of forest and the best fit with a linear model was for mangrove forests (slope = −3.296; *R* ^2^ = 0.346; Figure 2f). Tropical moist broadleaf forests (slope = −0.960, *R* ^2^ = 0.273; Figure 2a) and tropical savannas (slope = −2.180, *R* ^2^ = 0.211; Figure 2b) had the next best fits and a significant buffering effect on precipitation variability. For tropical moist broadleaf forest, this means that the CV of monthly precipitation is reduced by 53% if the fraction of precipitation originating from forest increases from 0 to 0.5. For tropical savannas, this effect is even stronger with a buffering effect of 67%. For mangroves, as the maximum fraction of precipitation originating from forest is 0.35 this buffering effect reaches 107%. This means that on average if tropical areas receive 50% of their precipitation from forest sources, they will have 68% lower variation in precipitation. More generally, and using the linear models, we found on average that for the biomes where forest had a significant buffering effect, if an area received 50% of its precipitation from forest, then the CV of monthly precipitation was reduced by 60% compared to a situation where none of the precipitation originates from forests.

Only two biomes exhibited an increase in precipitation variability with increasing fraction of precipitation originating from forest namely temperate coniferous forests (slope = 0.528, *R* ^2^ = 0.026; Figure 2j) and deserts (slope = 5.962, *R* ^2^ = 0.055; Figure 2n). We found no effect in boreal forests and tundra.

#### 3.2.2 Precipitation from non‐forest origin

**FIGURE 3:**
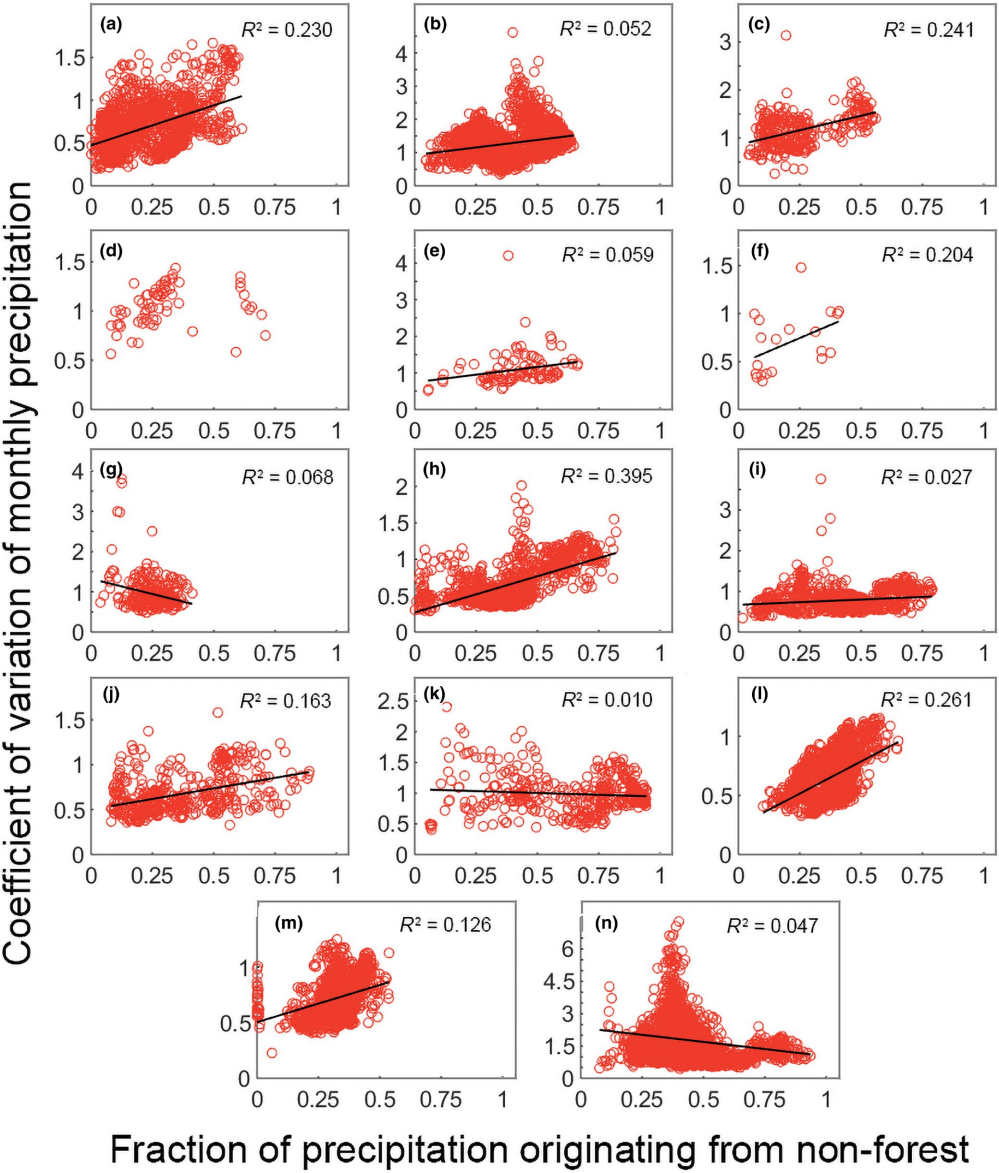
Linear regression of fraction of precipitation originating from non‐forest and coefficient of monthly precipitation variation. For plots where significant effects (*p* < 0.05) were detected we include a regression line and the *R* ^2^ value; we do not include a regression line when effects were non‐significant. (a) Tropical moist broadleaf forests; (b) Tropical savannas; (c) Tropical dry broadleaf forests; (d) Tropical coniferous forests; (e) Flooded Grasslands; (f) Mangroves; (g) Mediterranean forests; (h) Temperate broadleaf forests; (i) Temperate grasslands; (j) Temperate coniferous forests; (k) Montane grasslands; (l) Boreal forests; (m) Tundra; (n) Deserts

We found a significant positive effect of the fraction of precipitation from non‐forest origin on the CV of monthly precipitation for 10 out of the 14 analyzed biomes (Figure 3; Table S3). Put otherwise, when there is a higher fraction of precipitation coming from non‐forest, there is higher variability in monthly precipitation. Seven of these significant biomes had significant negative effects when considering precipitation originating from forest. The best fitting linear regressions occurred for the three broadleaf forests (tropical moist broadleaf forests slope = 0.934; *R* ^2 ^= 0.230; Figure 3a, tropical dry broadleaf forests slope = 1.213; *R* ^2^ = 0.241; Figure 3c, temperate broadleaf and mixed forests slope = 0.995; *R* ^2^ = 0.395; Figure 3h). The lack of buffering in these three biomes means that on average if areas receive 50% of their precipitation from non‐forest, there is a resulting increase in the CV of monthly precipitation of 66%. More generally, if an area within the 10 significant biomes receives 50% of precipitation from non‐forest, then there is an increase in 69% in the CV of monthly precipitation. We did find a buffering effect of non‐forest vegetation for Mediterranean forests (slope = −1.505; *R* ^2^ = 0.068; Figure 3g), montane grassland (slope = −0.121; *R* ^2^ = 0.010; Figure 3k) and deserts (slope = −1.321; *R* ^2^ = 0.048; Figure 3n); however, all had very weak fits. We found no significant effect for tropical coniferous forests.

#### 3.2.3 Precipitation from ocean origin

**FIGURE 4:**
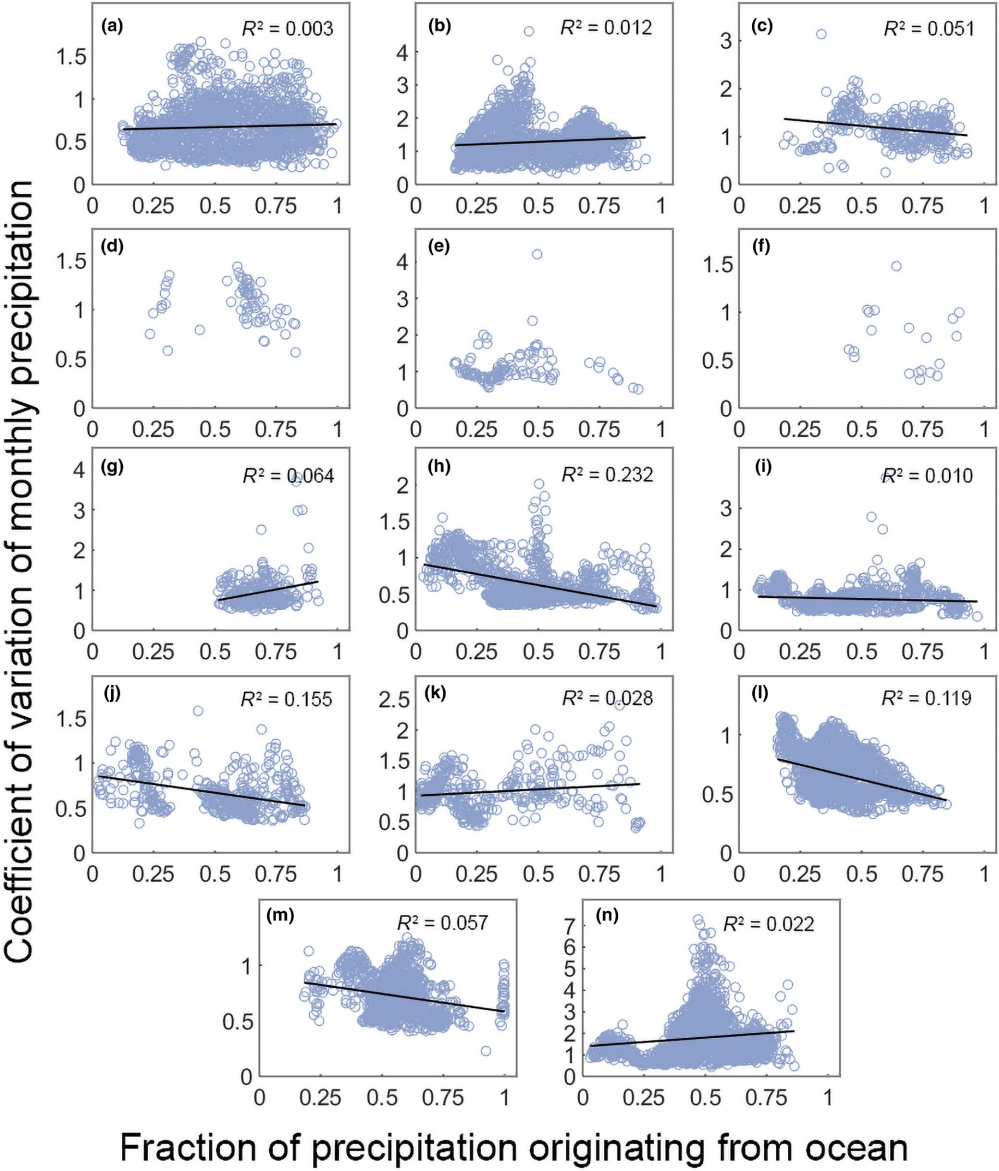
Linear regression of fraction of precipitation originating from ocean and coefficient of monthly precipitation variation. For plots where significant effects (*p* < 0.05) were detected we include a regression line and the *R* ^2^ value; we do not include a regression line when effects were non‐significant. (a) Tropical moist broadleaf forests; (b) Tropical savannas; (c) Tropical dry broadleaf forests; (d) Tropical coniferous forests; (e) Flooded Grasslands; (f) Mangroves; (g) Mediterranean forests; (h) Temperate broadleaf forests; (i) Temperate grasslands; (j) Temperate coniferous forests; (k) Montane grasslands; (l) Boreal forests; (m) Tundra; (n) Deserts

We found no clear pattern in the effect of precipitation fraction originating from ocean across the different biomes, with six biomes showing some buffering effect and the other five showing an increase in the CV of monthly precipitation; we also found that for three biomes here was no significant effect (Figure 4; Table S4). The strongest negative regressions occurred for temperate broadleaf forests (slope = −0.607; *R* ^2^ = 0.232; Figure 4h) and boreal forests (slope = −0.506; *R* ^2^ = 0.119; Figure 4l) while the strongest positive regression coefficients were for Mediterranean forests (slope = 1.128; *R* ^2^ = 0.064; Figure 4g) and deserts (slope = 0.8236; *R* ^2^ = 0.022; Figure 4n). We found that, on average, correlation coefficients for the majority of the biomes were lower than those identified for the fractions of precipitation originating from both forest and non‐forest.

## 4 DISCUSSION

Understanding the ability of forests to buffer precipitation variability elsewhere, is fundamental to better understand the hydrological cycle and the water‐climate regulatory ecosystem service it provides. We investigated whether forests buffer against monthly precipitation variability across the globe and we find that, indeed, these contributions are fundamental to reduce precipitation variability downwind in 10 out of the 14 global terrestrial biomes we examined. We demonstrate that if 50% of an area's precipitation originates from forests, there is a strong buffering effect with an average reduction of 60% in the CV of monthly precipitation. This buffering effect is not restricted to tropical forests only, but occurs across global biomes.

We find that the buffering effect of forests was strongest for tropical biomes, that is, tropical moist forests, tropical savannas and mangroves. For those three terrestrial biomes, we find that if 50% of a tropical area's precipitation originates from forests, then its CV of monthly precipitation is reduced by 68%. This can be explained by the inherent moisture recycling within tropical biomes, due to their dense and expansive forests and high evapotranspiration fluxes but also by contributions from elsewhere (Spracklen et al., 2018; Tuinenburg et al., 2020). Both the Amazon and Congo basins have been shown previously to recycle a high fraction of their evapotranspiration within their respective continents (van der Ent et al., 2010). Although Southeast Asia is home to 15% of all tropical rainforests (Estoque et al., 2019), the precipitation is more influenced by the surrounding ocean (Staal et al., 2020). As the evapotranspiration flux of tropical forests is relatively stable throughout the year, this reduces precipitation variability downwind. These results are in line with our hypothesized mechanism for the fraction of forest origin being more constant when forests are able to access to deep groundwater and maintain evapotranspiration, which is characteristic of tropical forests as they have longer rooting depths (Nepstad et al., 1994).

Of the other biomes for which we also detected a buffering effect, the fit was weaker. For instance, for temperate broadleaf forests (Figure 2h), the weak fit could be attributed to a low average forest cover of 34%, about half that found in tropical moist broadleaf forests (Table S1). The distance of atmospheric transport in many of the temperate regions is relatively short (Tuinenburg et al., 2020; van der Ent & Savenije, 2011) as a result low forest cover produces a much lower fraction of precipitation originating from forests. Furthermore, large proportions of temperate forests are in areas dominated by precipitation from non‐forest or ocean, such as in eastern Asia (Figure1b; Figure S1) and western Europe (Figure 1c; Figure S1). Therefore, the variation of precipitation will be more strongly influenced by these dominant sources. The fact that these biomes have vegetation with shallower rooting depths than their tropical counterparts (Fan et al., 2017) may also explain the weaker effect of these types of forests. Other biomes, for example, Mediterranean and temperate grasslands, had steeper decreases in the CV of monthly precipitation but lower fits of the regression model, in part because of the low forest cover (Table S1) and in part because larger fractions of precipitation come in from sources other than forest. We do know that in savannas and grasslands there are many species with deep roots, but these tend not to be dominant in the plant community (Canadell et al., 1996). Therefore, their effect becomes diluted in comparison to that observed for tropical forests, and the evapotranspiration fluxes are more variable (Zhang et al., 2016), explaining the lower effect for these grassy biomes.

Some biomes naturally occur in a small area globally, because of their specializations and adaptation to local climates and environmental conditions—for example, tropical coniferous forests, flooded grasslands and mangroves (Table S1). Therefore, despite an apparent lower “data sampling,” it actually includes all cells that globally are dominated by that biome so the results are in our understanding robust.

Interestingly, two biomes had a positive correlation between the fraction of precipitation originating from forest and CV of monthly precipitation, meaning that there was higher precipitation variability at high fractions of precipitation generated from forests. The first biome, deserts, is defined by low vegetation cover (<1% forest vegetation) and low and extremely variable monthly precipitation. The desert biomes are large and relatively remote from any large forested areas. This makes it highly unlikely for a consistent supply of atmospheric moisture originating from forests to reach and precipitate out over this biome. The areas with the highest CV of monthly precipitation were located in the Sahara Desert where the mean month precipitation of these cells was <1mm. The second biome was temperate coniferous forests; however, the effect we found was not very strong and had a weak fit. The temperate coniferous forest biome is concentrated on the western coast of North America and globally at high altitudes. These high altitudes experience high seasonality of evapotranspiration due to low temperatures (Falge et al., 2002), that is, at low temperatures as trees avoid frost damage by reducing photosynthesis they also reduce evapotranspiration fluxes. The final two biomes for which we did not find a significant effect, boreal forests and tundra, are also associated with below‐zero winter temperatures. These latter forested biomes also have shallow roots (Canadell et al., 1996). This is in line with our hypothesized mechanism. Furthermore, we believe that the biophysical limitations imposed by low temperatures supersede water accessibility effects.

Further supporting our hypothesis were the results for non‐forest land cover. In this analysis, we found a significant positive effect of non‐forested land area in the precipitationshed on the CV of monthly precipitation for 10 of the 14 biomes. Seven of these significant biomes also had a significant buffering effect when considering precipitation originating from forest. This indicates that when a high fraction of precipitation originates from non‐forest land cover, there is no buffering and variability of precipitation is high. This result further supports our hypothesis, as short vegetation and bare soil have less access to deep groundwater than forests and, as a result, have higher variability in evapotranspiration (e.g. Jackson et al., 1996; O'Connor et al., 2019). As evapotranspiration variability increases, in turn, there is higher variability in precipitation. We found that when 50% of an area's precipitation originates from non‐forest land, monthly precipitation variability increases by 69%. We also analyzed the fraction of precipitation originating from ocean. This is important as ocean is the largest contributor and the ultimate source of precipitation globally (Gimeno et al., 2012). However, no clear relationship exists between the fraction of precipitation originating from ocean and the variability of monthly precipitation. Only six of the 14 biomes had a significant negative correlation. Interestingly, the only tropical biome with a negative correlation was tropical dry broadleaf forests, which all occur close to the coast. All other tropical biomes, including mangroves, had either non‐significant, no or positively correlations. These tropical biomes have some of the highest fractions of forest cover (Hansen et al., 2013) and were all found to have a negative correlation between the CV of monthly precipitation and the fraction of forest precipitation.

Our study is driven by climate reanalysis data between 2000 and 2016. With the current structure of the model, we are unable to make accurate predictions of how the future climate may influence moisture recycling and whether the contribution from forests may change. Climate change is predicted to increase global temperatures and increase the occurrence and severity of climate extremes (both droughts and floods; Trenberth, 2011). These changes, combined with increasing atmospheric CO_2_ concentrations, may have several impacts on the hydrological system. Remote sensing has revealed a global greening of the Earth's surface as a result of rising CO_2_ (Zhu et al., 2016). The increased vegetation may lead to increases in evapotranspiration. However, the rising CO_2_ reduces the density and diameter of leaf stomata, in turn, reducing transpiration rates (Lammertsma et al., 2011). The probable reduction in continental moisture recycling will increase the dependance of precipitation regimes on oceanic evaporation (Findell et al., 2019). While moisture recycling may increase in wet regions (Zeng et al., 2018), dry areas may become drier or get longer dry seasons (Sherwood & Fu, 2014; Trenberth, 2011). Warming may shift the current boundaries of biomes as species tolerances for changing temperatures reaches their limits (Gonzalez et al., 2010). The combined effect of tropical deforestation and climate change may trigger positive feedbacks that accelerate a breakdown of the moisture recycling system and release stored CO_2_ (Hoffmann et al., 2003; Lovejoy & Nobre, 2018). As the moisture recycling system includes cascades, deforestation impacts may result in repeated losses of precipitation as the cascade is broken (Staal et al., 2018; Zemp et al., 2017).

Previous studies have already shown that forest cover leads to a higher magnitude of moisture recycling (Jasechko et al., 2013; Spracklen et al., 2012, 2018). Our results show that forests are also important to buffer precipitation variability. The question remains whether afforestation could be used to decrease precipitation variability. Theoretically, high forest cover can increase precipitation over an area by drawing evaporated moisture in from the oceans (Sheil, 2014). To be effective, continuous forest is needed between the target sink and the coast (Makarieva & Gorshkov, 2007) to maintain the flow of water inland. In our analysis, we found some evidence of the relationship between forest cover at the precipitation site and CV of precipitation (Figure 2; Figure S2). However, we believe that this effect is not driving the relationships that we identified between precipitation originating from forest and CV of precipitation. Our model presented in this paper could be applied to study a specific area's precipitationshed where dominant upwind areas are targeted for reforestation. However, these efforts will be strongly dependent on geographic location as not every area has a precipitationshed over land. Further, there are large differences between the proportion of land‐to‐ocean flows between the Northern and Southern Hemisphere. Large‐scale circulatory patterns drive atmospheric transport and as a result will carry evaporated water from oceans over continents in some areas while drawing water from evapotranspiration toward the oceans in another. In addition, changes in land surface can have different effects on atmospheric circulation, which may influence atmospheric cycling (Makarieva et al., 2013; Vergopolan & Fisher, 2016). There has been a net increase in global forest cover in recent decades, although the changes in forest cover vary geographically (Song et al., 2018). In temperate and boreal forest regions, there has been a net gain, whereas, in the tropics, there has been a net loss (Song et al., 2018). As forest cover in temperate regions is increasing, we expect an increase in forest moisture recycling and more stable monthly precipitation in the temperate zone. The increase in forest cover is dispersed over a large area; therefore, the effect on precipitation variability may only be marginal for any given location. In addition, as we did not find a significant effect in boreal forests, it is not clear whether increases in forest cover will buffer monthly precipitation there. Ongoing forest cover loss is largely concentrated in the tropics (Hansen et al., 2013), so we expect the largest increases in precipitation variability to occur in these regions. Indeed, deforestation in the Amazon has already been linked with a lengthening of the dry season (Butt et al., 2011; Debortoli et al., 2017; Leite‐Filho et al., 2019). More generally, our findings support an important role of forests in buffering precipitation downwind. The importance of these findings relates to the ability of moisture recycling to regulate the climate system, which can become unbalanced if this regulating ecosystem service is removed by, for instance, deforestation or replacement of forest by other vegetation. Furthermore, the importance of this mechanism is also relevant to maintain other processes, such as food production (Mu et al., 2021), and highlights the tight connections between forests and other processes and ecosystem services. Thus, our results can be used to understand the coupled effects of cutting forests in one location on to “downwind” processes at regional and also global levels.

## Supporting information

Supplementary Material

## Data Availability

The data that support the findings of this study are available from the corresponding author upon reasonable request.
