# Supplementary material for: Forests buffer against variations in precipitation
Source: Glob Chang Biol. 2021 Jul 28;27(19):4686–96. doi: 10.1111/gcb.15763 (PMC8457185; doi:10.1111/gcb.15763)
Supplement: Supplementary file 1 — Supplementary Material [file GCB-27-4686-s001.pdf]

Appendix

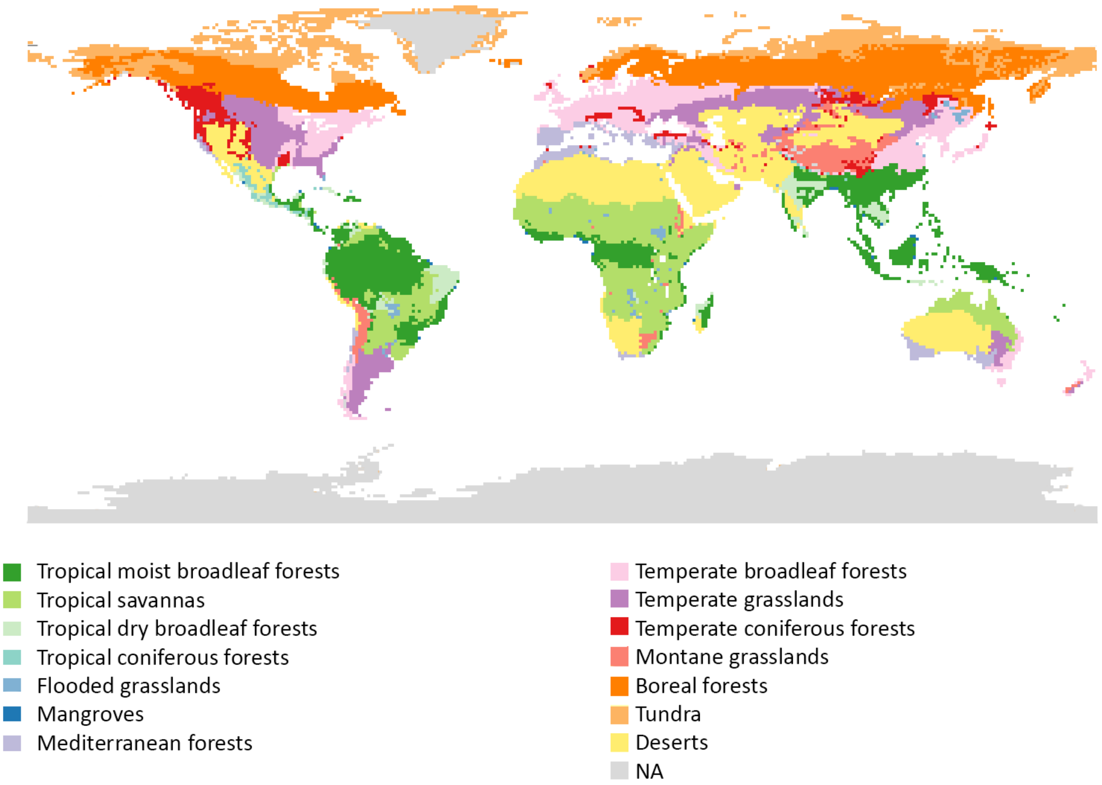

Figure S1. Depicting the global distribution of each biome range included in the study. Data downloaded from ecoregions2017appspot.com

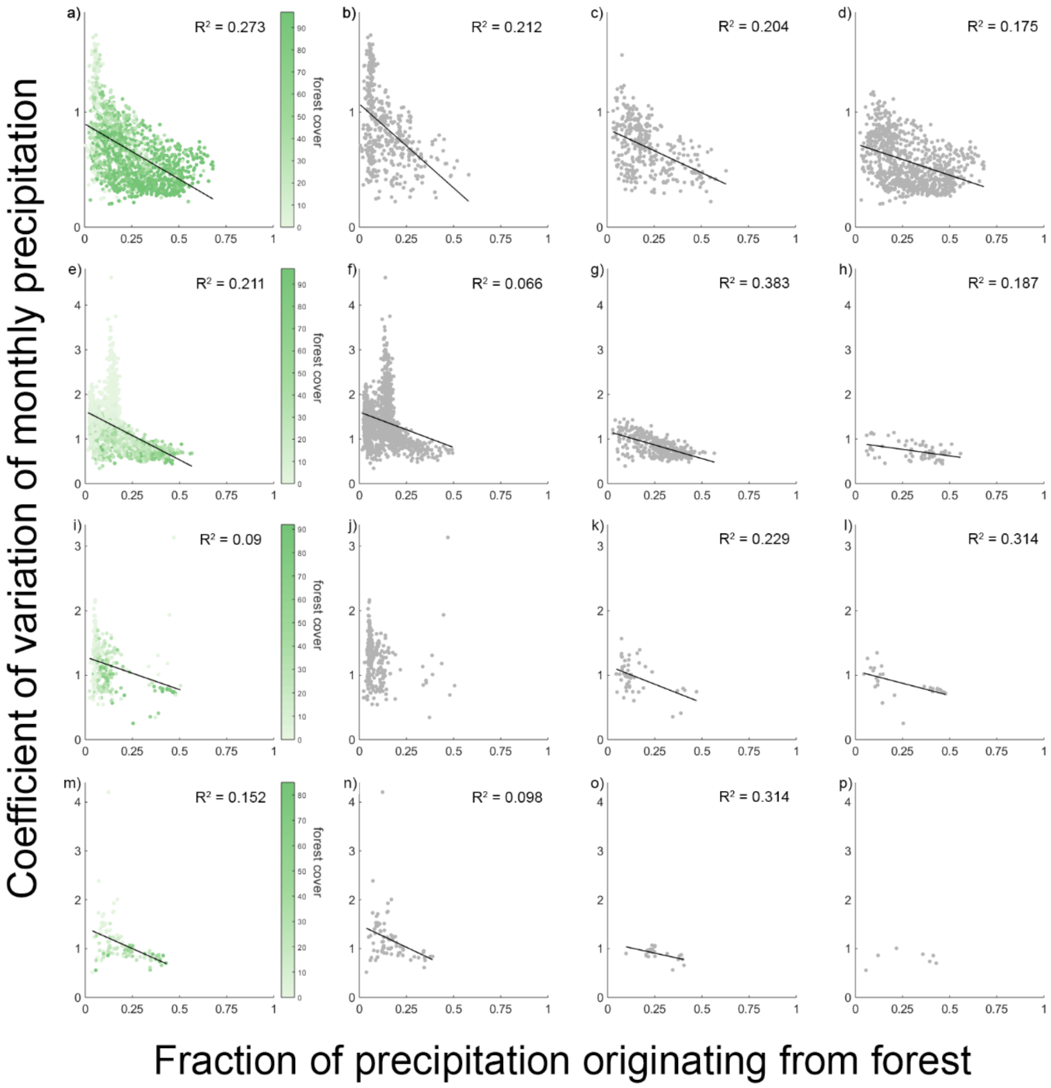

Figure S1 Tropical moist forest, CV of precipitation as a function of precipitation originating from forest. a,e,i,m) color shading represents the fraction of forest cover. b,f,j,n) locations with <30% forest cover, c,g,k,o) locations with >30% forest cover and <60% forest cover d,h,l,p) locations with >60% forest cover. a-d) Tropical moist broadleaf forests; e-h) Tropical savannas; i-l) Tropical dry broadleaf forests; m-p) Flooded Grasslands

Table S1. Summary of the 14 biomes used in the study.

Biomes	No. Cells	Mean Forest Cover ± SD		Mean Precipitation mm.month^{-1} ± SD		Mean Fraction of precipitation originating from forest ± SD	
Tropical moist broadleaf forests	1625	57.50	7.26	173.05	106.38	23.05	9.90
Tropical savannas	1806	17.47	4.65	68.66	70.44	16.09	12.25
Tropical dry broadleaf forests	328	21.22	5.27	94.14	99.90	11.52	10.69
Tropical coniferous forests	67	22.86	6.12	89.01	88.76	9.20	6.59
Flooded Grasslands	103	23.76	6.28	69.94	67.81	19.20	11.56
Mangroves	30	26.35	5.32	121.45	74.05	11.92	8.61
Mediterranean forests	324	8.43	2.95	37.71	31.76	6.77	9.41
Temperate broadleaf forests	1434	33.64	7.68	73.45	44.89	15.55	8.97
Temperate grasslands	1196	11.59	4.11	44.34	32.29	12.78	9.74
Temperate coniferous forests	461	38.99	7.37	81.33	51.43	14.48	9.26
Montane grasslands	451	3.64	1.20	56.01	50.47	7.52	6.15
Boreal forests	2516	38.83	8.20	47.98	30.23	20.10	11.38
Tundra	2010	6.16	2.96	31.40	21.71	11.26	10.57
Deserts	2496	0.72	0.38	14.53	18.32	9.20	12.95

Table S2 Linear regression statistical ouput from figure 2. Fraction of precipitation from forest, coefficient of variation of monthly precipitation

**Forest Origin**	**Intercept**	**Estimate**	**SE**	**$T_{statistic}$**	**$F_{statistic}$**	**p-value**	**R2**
Tropical moist broadleaf forests	0.899	-0.960	0.039	-24.464	598.481	0.000	0.273
Tropical savannas	1.625	-2.180	0.099	-21.913	480.174	0.000	0.211
Tropical dry broadleaf forests	1.283	-1.015	0.181	-5.615	31.531	0.000	0.090
Tropical coniferous forests	1.352	-3.195	0.908	-3.520	12.390	0.001	0.162
Flooded Grasslands	1.427	-1.712	0.403	-4.248	18.044	0.000	0.152
Mangroves	1.075	-3.296	1.069	-3.084	9.513	0.006	0.346
Mediterranean forests	1.084	-2.122	0.768	-2.761	7.625	0.006	0.025
Temperate broadleaf forests	0.681	-0.242	0.108	-2.255	5.085	0.024	0.004
Temperate grasslands	0.865	-0.653	0.133	-4.917	24.173	0.000	0.020
Temperate conifer forests	0.595	0.528	0.150	3.516	12.362	0.000	0.026
Montane grasslands	1.058	-0.986	0.200	-4.939	24.393	0.000	0.052
Boreal forests	0.673	-0.048	0.071	-0.680	0.463	0.496	0.000
Tundra	0.737	-0.106	0.086	-1.232	1.518	0.218	0.001
Deserts	1.237	5.962	0.497	11.986	143.659	0.000	0.055

Table S3 Linear regression statistical ouput from figure 4. Fraction of precipitation from non-forest, coefficient of variation of monthly precipitation

**Non-Forest Origin**	Intercept	Estimate	SE	$T_{statistic}$	$F_{statistic}$	p-value	R2
Tropical moist broadleaf forests	0.473	0.934	0.043	21.793	474.948	0.000	0.230
Tropical savannas	0.926	0.910	0.091	9.954	99.077	0.000	0.052
Tropical dry broadleaf forests	0.855	1.213	0.120	10.091	101.829	0.000	0.241
Tropical coniferous forests	0.974	0.268	0.150	1.786	3.188	0.079	0.047
Flooded Grasslands	0.742	0.843	0.336	2.509	6.293	0.014	0.059
Mangroves	0.472	1.090	0.507	2.149	4.617	0.046	0.204
Mediterranean forests	1.322	-1.505	0.320	-4.699	22.078	0.000	0.068
Temperate broadleaf forests	0.269	0.995	0.033	30.047	902.792	0.000	0.395
Temperate grasslands	0.669	0.259	0.045	5.793	33.555	0.000	0.027
Temperate coniferous forests	0.502	0.470	0.050	9.425	88.840	0.000	0.163
Montane grasslands	1.064	-0.121	0.057	-2.107	4.439	0.036	0.010
Boreal forests	0.244	1.089	0.037	29.631	877.995	0.000	0.261
Tundra	0.505	0.668	0.042	15.740	247.740	0.000	0.126
Deserts	2.348	-1.321	0.119	-11.089	122.958	0.000	0.047

Table S4 Linear regression statistical ouput from Figure 4. Fraction of precipitation from ocean, coefficient of variation of monthly precipitation

**Ocean Origin**	Intercept	Estimate	SE	$T_{statistic}$	$F_{statistic}$	p-value	R2
Tropical moist broadleaf forests	0.637	0.068	0.033	2.056	4.229	0.040	0.003
Tropical savannas	1.136	0.302	0.065	4.645	21.575	0.000	0.012
Tropical dry broadleaf forests	1.454	-0.460	0.111	-4.141	17.146	0.000	0.051
Tropical coniferous forests	1.188	-0.219	0.162	-1.350	1.821	0.182	0.028
Flooded Grasslands	1.044	0.143	0.273	0.524	0.275	0.601	0.003
Mangroves	0.950	-0.377	0.496	-0.759	0.576	0.458	0.031
Mediterranean forests	0.174	1.128	0.248	4.551	20.708	0.000	0.064
Temperate broadleaf forests	0.927	-0.607	0.030	-20.428	417.322	0.000	0.232
Temperate grasslands	0.839	-0.132	0.038	-3.467	12.021	0.001	0.010
Temperate coniferous forests	0.863	-0.388	0.043	-9.119	83.152	0.000	0.155
Montane grasslands	0.929	0.210	0.058	3.593	12.913	0.000	0.028
Boreal forests	0.873	-0.505	0.028	-18.335	336.185	0.000	0.119
Tundra	0.902	-0.319	0.031	-10.240	104.853	0.000	0.057
Deserts	1.388	0.824	0.111	7.449	55.482	0.000	0.022
